# Feedback, both Fast and Slow: How the Retina Deals with Redundancy in Space and Time

**DOI:** 10.1371/journal.pbio.1001865

**Published:** 2014-05-20

**Authors:** Richard Robinson

**Affiliations:** Freelance Science Writer, Sherborn, Massachusetts, United States of America

[Fig pbio-1001865-g001]By the time visual information leaves the retina for the brain, it has undergone several rounds of processing. Most prominently, that information has been filtered to amplify differences in contrast, between either two points in space or two points in time. The initial step in that amplification is carried out by horizontal cells, whose job is not only to receive signals from a group of adjacent photoreceptor cells (rods or cones) but also to send inhibitory feedback signals back to the photoreceptors, turning down the weakest outputs and allowing only the strongest to get through.

**Figure pbio-1001865-g001:**
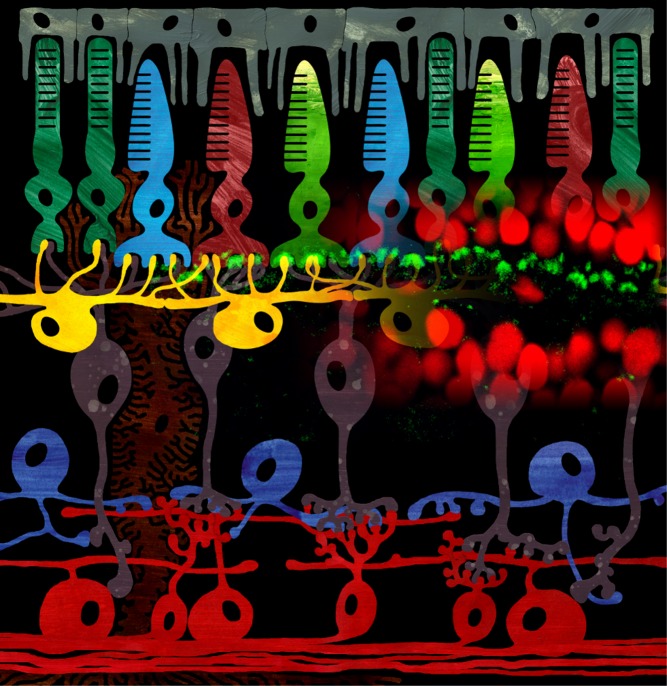
Schematic drawing of the vertebrate retina combined with the immunolocalization of the ecto-ATPase NDTPdase1 (green). *Image Credit*: *Rozan Vroman created the image, Jan Klooster provided the immunocytochemical picture.*

This much has been known for several decades, but the cell physiological details of the feedback mechanism have been controversial. Part of the puzzle has been the paradoxical requirement for both very fast feedback, needed to highlight spatial differences, and slower feedback, needed to process temporal ones. In this issue of *PLOS Biology*, Rozan Vroman, Maarten Kamermans, and colleagues resolve this apparent paradox: they show that horizontal cells in the goldfish retina employ two simultaneous feedback mechanisms, the slower of which relies on a newly described mechanism, creating an extracellular buffer by secreting and hydrolyzing ATP.

Horizontal cells affect the activity of cone cells by changing the rate of flow of positively charged calcium ions from the extracellular space through calcium channels into the cone cell. The authors measured that current within individual cone–horizontal cell synapses, and found that the curve reflecting the current change was best described as the sum of two exponential functions, one representing an initial fast increase and the other a slower one.

The fast component was very fast indeed, inconsistent with the inevitable delay imposed when cells are separated by a synaptic gap. Instead, the authors show that connexin hemichannels on the horizontal cell membrane were involved. These channels allowed current to flow into the horizontal cell, increasing the negative charge of the intercellular space and increasing the flow of calcium into the cone cell, locally depolarizing it. This direct (“ephaptic”) connection makes the synapse among the fastest of all known inhibitory synapses.

But it was the slow component that held the most surprises. The authors noted that light-induced feedback responses of horizontal cells can be completely blocked by carbenoxolone, which antagonizes not only connexin but also another channel, called pannexin 1 (Panx1). Panx1 is also present in the synapse and is known to mediate release of ATP. The authors showed that depolarized horizontal cells released ATP, an effect that could be blocked by addition of a specific Panx1 antagonist, probenecid.

When hydrolyzed, ATP produces phosphates and H+ ions (protons), which could acidify the intercellular space as well as buffer it against pH changes. The authors used immunolabeling to show that an ATP hydrolase was present on the exterior surface of the horizontal cell, and that blocking this enzyme reduced the slow component of the feedback response. Artificial alkalization of the synaptic space had the same effect. The ATP that is released via Panx1 is thus hydrolyzed and leads to slight acidification of the synaptic cleft, which inhibits presynaptic calcium channels and inhibits the neurotransmitter release of photoreceptors.

The combination of two feedback components with different speeds allows the horizontal cell–cone system to respond to both spatial and temporal variation, the authors argue. Spatially, the role of the horizontal cell is, in effect, to subtract the average level of activity in multiple adjacent cones from the output of each, allowing only the most prominent signals through. That subtraction must happen very fast for a moving signal to be processed correctly—and the ephaptic component is about as fast as it gets. Temporally, the horizontal cell must subtract out long-lasting stimulation from the cone output, which by definition requires a slower mechanism, provided by the Panx1–ATPase system.

Although these experiments were done in fish, mice also express Panx1 at their horizontal cell synapses, suggesting the mechanism likely operates in mammals as well. The authors speculate that variations in the relative activity of the fast and slow mechanisms may contribute to interspecies differences in responsiveness to spatial versus temporal stimuli. They also point out that Panx1 is widely distributed in the brain, suggesting that an ATP pH effect may contribute to feedback systems in other types of neurons as well.

**Vroman R, Klaassen LJ, Howlett MHC, Cenedese V, Klooster J, et al. (2014) Extracellular ATP Hydrolysis Inhibits Synaptic Transmission by Increasing pH Buffering in the Synaptic Cleft.** doi:10.1371/journal.pbio.1001864

